# Cytotoxic activity of *Androctonus australis hector* venom and its toxic fractions on human lung cancer cell line

**DOI:** 10.1186/s40409-016-0085-4

**Published:** 2016-10-22

**Authors:** Louisa Béchohra, Fatima Laraba-Djebari, Djelila Hammoudi-Triki

**Affiliations:** USTHB, Faculty of Biological Sciences, Laboratory of cellular and Molecular Biology, BP32, El Alia, Bab Ezzouar, 16111 Algiers, Algeria

**Keywords:** Aah venom, cytotoxicity, F3 fraction, Apoptosis, Lung cancer cells, Oxidative stress

## Abstract

**Background:**

Several studies have showed that animal venoms are a source of bioactive compounds that may inhibit the growth of cancer cells, which makes them useful agents for therapeutic applications. Recently, it was established that venom toxins from scorpions induced cytotoxic, antiproliferative and apoptogenic effects on cancer cells. Therefore, the present study aims to investigate the cytotoxic activity of *Androctonus australis hector* (Aah) scorpion venom and its toxic fractions (FtoxG-50 and F3) on NCI-H358 human lung cancer cells.

**Methods:**

The cytotoxic and antiproliferative activities were estimated using MTT assay, lactate dehydrogenase release and clonogenic assays. Apoptosis was evaluated by Hoechst 33258 staining, DNA fragmentation assay and caspase-3 activity. Oxidative stress was analyzed by reactive oxygen species, nitric oxide, malondialdehyde and protein carbonyl levels along with assessment of antioxidant status. In addition, alteration of mitochondrial membrane potential was analyzed by JC1 fluorescent dye.

**Results:**

The present findings showed that F3 fraction was more cytotoxic towards NCI-H358 lung cancer cells with an IC_50_ of 27.05 ± 0.70 μg/mL than venom alone (396.60 ± 1.33 μg/mL) and its toxic fraction FtoxG-50 (45.86 ± 0.91 μg/mL). Nevertheless, F3 fraction was not cytotoxic at these concentrations on normal human lung fibroblast MRC-5 cells. Inhibition of NCI-H358 cell proliferation after F3 fraction exposure occurred mainly by apoptosis as evidenced by damaged nuclei, significant DNA fragmentation level and caspase-3 activation in a dose dependent manner. Moreover, F3 fraction enhanced oxidative and nitrosative stress biomarkers and dissipated mitochondrial membrane potential in lung cancer cells along with significant depletion in cellular enzymatic and non-enzymatic antioxidants. Further, the apoptosis induced by F3 fraction was markedly prevented by the antioxidant N-acetylcysteine (NAC) suggesting the potential mechanism of oxidative stress.

**Conclusion:**

These findings suggest that F3 fraction could induce apoptosis in lung cancer cells through involvement of oxidative stress and mitochondrial dysfunction. Hence, these properties make F3 fraction a promising candidate for development of new anticancer agents.

## Background

Cancer represents a major public health problem and is a leading cause of death worldwide. As reported by International Agency for Research on Cancer (IARC), lung, stomach, liver, colon and breast cancers are known to be the highest contributors to cancer mortality each year. Despite the favorable advancement in development of chemotherapeutic drugs, the lack of selectivity to tumor cells and toxic side effects limit their effectiveness [1, 2]. Biomedical research using natural resources has established that animal venoms and toxins present a great potential against various pathophysiological conditions. Venoms of animal species such as snakes, scorpions, bees, spiders, jellyfish and sea anemones are a complex of bioactive molecules that display a plethora of molecular targets and functions [3–7]. Scorpions venoms have been used as traditional therapies in various pathophysiological conditions in Asia and Africa [2]. These venoms also contain several pharmacologically active components that could elicit great interest as a source of proteins and peptides for the design and the development of new drugs with antimicrobial, antiparasitic, analgesic, immunosuppressing and anticancer activities [1, 3, 5].

Recently, several investigations demonstrated that venoms and toxins of some species of scorpions, especially those of the Buthidae family, induce cytotoxic antiproliferative effects and apoptosis on cancer cells in vitro and in vivo [8–12]. Cell growth inhibition is essentially due to proteins and peptides isolated from these venoms, some of them are explored in phase I and phase II clinical trials [3]. Scorpion venom peptides can induce their anticancer effect by blockage of specific ion channels or bind to specific targets in the membranes of cancer cells such as chlorotoxin – a peptide isolated from *Leiurus quinquestriatus* [13] – or most importantly by triggering extrinsinc or intrinsinc apoptosis such as bengalin and neopladines (1 and 2) – peptides isolated from *Heterometrus bengalensis* Koch and *Tityus discrepans* respectively [14, 15]. The peptides purified from scorpion venoms were also able to exert a dual function with antimicrobial and antitumor activities or analgesic and antitumor activities such as BmK AGAP-SYPU2, TsAP-1 and TsAP-2 respectively [16, 17].

Scorpion venoms that belong to the Buthidae family present a complex composition with toxic and non-toxic fractions. The non-toxic fraction is a mixture of mucopolysaccharides, hyaluronidases, phospholipases and enzymes inhibitors. The lethal effects of scorpion venoms were largely attributed to the toxic fraction which consists mainly in highly specific neurotoxins to ion channels (sodium, potassium, calcium or chloride) of excitable and non excitable cells [18]. *Androctonus australis hector* (Aah) scorpion is the most endemic species from North Africa belonging to Buthidae family [19]. Typical manifestations of Aah scorpion envenomation are cardiac dysfunction, systemic inflammatory response syndrome, pulmonary edema and respiratory failure [20]. Three fractions were isolated from this venom by gel filtration. The non-toxic fraction was called F1. The two in vivo toxic fractions that potentiate Aah venom pathogenesis were FtoxG50 that contains toxins of 7 kDa that mainly target sodium voltage gated channels (Na_v_), and the latest eluted toxic fraction F3 that contains neurotoxins with small molecular weight (~3 and 4 kDa) active on potassium voltage gated channels (K_v_) [21, 22].

In a recent study, our research team demonstrated the ability of Aah venom and its non-toxic fraction 1 (F1) to inhibit proliferation of early stage hepatocarcinoma induced in vivo by Fumonisin B1 mycotoxin [23]. In the same context, the present study was carried out to investigate the antiproliferative and cytotoxic induction capacity of Aah crude venom and its toxic fractions (FtoxG-50 and F3) on cancer cells in vitro.

## Methods

### Chemicals

The following chemicals were purchased from Sigma Aldrich (USA): Roswell Park Memorial Institute 1640 (RPMI 1640), Dulbecco’s modified Eagle’s medium (DMEM), fetal bovine serum (FBS), N-(1-napthyl)-ethylenediamine dihydrochloride, sulfanilamide, sodium nitrite, 3-(4, 5 dimethylthiazol-2-yl)-2,5-diphenyl-tetrazolium bromide (MTT), 5,5′-dithio bis (2-N benzoic acid) (DTNB), 1,1,3,3-tetraethoxy-propane (TEP), 2, 7-dichlorodihydrofluorescein diacetate (DCFDA-H2), 5,5,6,6-tetrachloro-1,1,3,3-tetraethylbenzimidazolyl-carbocyanine iodide (JC1), Hoechst 33258 (HO), 2,4-dinitrophenylhydrazine (DNPH), diphenylamine (DPA), dimethylsulfoxide (DMSO), methionine, N-acetylcysteine (NAC), nitroblue terazolium (NBT), riboflavin, and thiobarbituric acid (TBA). Triton X-100, potassium dichromate, trichloroacetic acid (TCA) and glacial acetic acid were purchased from Merck (Germany). Cisplatin was purchased from Mylan (France).

### Cell lines and cell culture

The following cell lines were purchased from American Type Culture Collection (ATCC, Manassas, VA): HeLa (cervix adenocarcinoma), Hep2 (laryngeal carcinoma cell line), MCF7 (human breast cancer cells), NCI-H358 (human lung adenocarcinoma cells) and MRC5 (normal human lung fibroblast). HeLa, Hep2, MCF7 and MRC5 cells were cultured in Dulbecco’s modified Eagle’s medium (DMEM), supplemented with 10 % heat inactivated FBS, 2 mM L-glutamine, penicillin (100 U/mL) and streptomycin (100 μg/mL). The NCI-H358 cell line was maintained in RPMI-1640 supplemented with 10 % (v/v) FBS, penicillin (100 U/mL), streptomycin (100 μg/mL) and 1 % of non-essential amino acid. All cell lines were incubated in the presence of 5 % CO_2_/95 % air at 37 °C.

### Scorpion venom and its toxic fractions

Aah venom was obtained from Pasteur Institute of Algeria and solubilized in sterile double distilled water. After centrifugation at 10,000 × *g* for 15 min at 4 °C, supernatant was dissolved in phosphate buffer serum (PBS) and then passed through nitrocellulose filter (Millipore 0.45 μm) and protein content was determined by Bradford method [24]. Aah venom toxic fractions (FtoxG-50) and fraction 3 (F3) were isolated from the venom by gel filtration through Sephadex G50 column as previously reported [21].

### Cell viability assay

Cells were seeded into 96-well culture plates at the concentration of 1 × 10^4^ cells per well and incubated overnight at 37 °C in 5 % CO_2_. Media was removed and cells were exposed to increasing concentrations of crude venom (50–500 μg/mL) or its components, FtoxG-50 and F3 fractions (5–50 μg/mL) for 24 h. The medium was discarded and replaced by MTT solution in 100 μL of fresh medium (0.5 mg/mL) and incubated for an additional 4 h at 37 °C, 5 % CO_2_. Subsequently, the medium was aspirated, and the resulting purple formazan crystals were dissolved in DMSO [25]. The plate was shaken for 10 min, and the reaction product was measured with Absorbance Microplate Reader (Infinite® Pro200 TECAN, Switzerland) at 550 nm. Cell viability was expressed as a percent of the control culture value and the IC_50_ was calculated from nonlinear regression using the program GraphPad Prism 5.0 software (GraphPad Software, USA).

### Lactate dehydrogenase (LDH) release assay

NCI-H358 cells were seeded in a 96-well plate at density of 2 × 10^4^ cells per well in culture medium. After overnight incubation, medium was replaced and cells were exposed to varying concentrations of Aah venom and its components (FtoxG-50 and F3) for 24 h. LDH activity was measured in the cell lysates and cell supernatants using LDH commercial kit (Spinreact, Spain) in accordance with the manufacturer’s instructions. Percentage of LDH release was calculated as the activity of LDH in the medium versus total LDH activity in the cells (medium and cell lysates).

### Clonogenic assay

The clonogenic assay is an in vitro survival assay of cells based on the ability of a single cell to grow into a colony. In brief, cells were plated in 12-well plates at a density of 500 cells/well for 24 h, prior to the addition of various concentrations of F3 fraction (½ IC_50_, IC_50_ and 2 IC_50_). After 24 h of treatment, fraction F3-containing medium was removed and replaced with complete growth medium. Medium was changed every three days for 8 to 10 days until visible colonies were formed. Colonies were simultaneously fixed and stained with 0.5 % crystal violet (in methanol/water, 1:1). Individually stained colonies in each well were counted and the colony surviving fraction was calculated as follows: (plating efficiency (PE) of treated cells/PE of control cells) × 100 %, where PE is the number of formed colonies divided by the number of plated cells.

### Morphological assessment

Cells were seeded into 6-well plates at the density of 5 × 10^5^ cells/well and allowed to adhere overnight. After 24 h of treatment with the F3 fraction (½ IC_50_, IC_50_ and 2 IC_50_), morphological characteristics were observed and the images were captured under an inverted phase-contrast microscope (Hund Wetzlar, Germany) at 100× magnification.

### Apoptosis detection with Hoechst 33258

Hoechst 33258 staining was used to detect the apoptotic cells including nuclear chromatin condensation and apoptotic bodies [26]. Cells were subcultured on sterile glass cover slips in 6-well plates. Cells were incubated with different concentrations of F3 fraction (½ IC_50_, IC_50_ and 2 IC_50_) for 24 h and were pretreated or not with antioxidant NAC (3 mM for 1 h). All cells were then washed with PBS, fixed with 4 % formaldehyde for 10 min, washed again twice with PBS and stained with Hoechst 33258 (10 μg/mL) for 20 min in dark at room temperature. Cells were examined under fluorescence microscopy (Zeiss Axioplan, Germany) at 400× magnification and cells with bright color, condensed or fragmented nuclei were considered as apoptotic. At least 200 cells from randomly selected fields were counted for each data point using Image J software.

### DNA fragmentation estimation

DNA fragmentation was measured by diphenylamine (DPA) assay according to the method described by Bouaziz et al. [27]. Cells were incubated with ½ IC_50_, IC_50_ and 2 IC_50_ concentrations of F3 fraction and IC_50_ of cisplatin for 24 h at 37 °C. The cells were harvested and centrifuged at 3000 × *g*, and then the cell pellet was resuspended in TTE lysis solution (Triton X-100 0.2 %, Tris-HCl 10 mM, EDTA 1 mM). After centrifugation (5000 × *g*, 30 min), the pellet with the intact DNA was resuspended in 200 μL of lysis buffer and the supernatant with fragmented DNA was transferred into new conical tubes. Trichloro-acetic acid (TCA 25 %) was added to each supernatant and resuspended pellet followed by incubation overnight at 4 °C. After centrifugation (5000 × *g*, 10 min), 160 μL of 5 % TCA was added to each tube and incubated for 15 min at 90 °C. Solution of 320 μL of DPA was added to the sample, followed by incubation overnight at room temperature. The optical density was determined at 600 nm and percentage of DNA fragmentation was calculated as follows:

$$ \%\ \mathrm{fragmented}\ \mathrm{D}\mathrm{N}\mathrm{A}=\left[\frac{\mathrm{OD}\ \mathrm{supernatant}}{\mathrm{OD}\ \mathrm{supernatant} + \mathrm{O}\mathrm{D}\ \mathrm{pellet}}\right]\times 100 $$

### Caspase-3 activity assay

Activity of caspase-3 was performed using CASP-3-C colorimetric assay kit (Sigma-Aldrich, USA), according to the manufacturer’s instructions. Cell lysates obtained from F3 fraction-treated or -untreated NCI-H358 cells were incubated with 2 mM Caspase-3 substrate (Ac-DEVD-pNA) and simultaneously incubated with or without the caspase-3 inhibitor Ac-DEVD-CHO (200 μM) for 1.5 h at 37 °C. Cisplatin was considered the positive control. The enzyme activity was followed by yellow color development at 405 nm and the results were calculated using p-nitroaniline calibration curve.

### Reactive oxygen species (ROS) assay

Intracellular ROS production was assayed using 2, 7-dichlorodihydrofluorescein diacetate (DCFH-DA), as described by Wang et al. [28]. NCI-H358 cells were seeded (2 × 10^4^ cells per well) in black 96-well plates and incubated for 24 h. After 1, 4 and 24 h of exposure to F3 fraction (½ IC_50_, IC_50_ and 2 IC_50_) with or without antioxidant (NAC)-pretreatment (3 mM for 1 h), media were removed, and 10 μM DCFH-DA in fresh media was added. Cells were incubated for 30 min at 37 °C and 5 % CO_2_ in the dark. Subsequently, cells were washed twice with PBS and the fluorescence intensity of DCF was measured at excitation/emission wavelength of 485/530 nm, respectively, with Victor *X*2 Multilabel Plate Reader (Perkin Elmer, USA). Values were normalized to the percentage in untreated control groups.

### Nitric oxide (NO) production assay

Nitrite accumulation was measured in cell culture supernatants using the Griess reagent [29]. Briefly, the cells were seeded into 96-well plates at a density of 2 × 10^4^ cells per well and incubated overnight. Thereafter, media was discarded and cells were exposed to medium containing increasing concentrations of F3 fraction (½ IC_50_, IC_50_ and 2 IC_50_). After 24 h, media from each well was transferred to fresh tube. Following centrifugation at 500 × *g* for 5 min at 4 °C, 100 μL of the supernatant was transferred into a fresh 96-well plate and mixed with an equal volume of Griess reagent at room temperature for 10 min. The absorbance was measured at 540 nm. The nitrite concentration was calculated from sodium nitrite standard curve and expressed as μM/mL.

### Lipid peroxidation (MDA) assay

Malondialdehyde (MDA) quantification was conducted according to the method of Ohkawa et al. [30]. Briefly, 100 μL of cell lysates obtained after cells incubation with lysis buffer [20 mM Tris-HCl (pH 7.5), 150 mM NaCl, 1 mM EDTA and 1 % Triton X-100] were mixed with 0.2 mL of 8.1 % SDS, 1.5 mL of 20 % acetic acid (pH 3.5) and 1.5 mL of 0.8 % thiobarbituric acid in test tubes. The mixture was heated at 95 °C for 120 min and the absorbance was measured at 532 nm. The concentrations of MDA were calculated using 1,1,3,3-tetraethoxy-propane (TEP) as a reference standard and expressed as nM of MDA formed per mg of protein.

### Protein carbonyl assay

Quantification of carbonyl content was determined by the derivatization of protein carbonyl groups with 2,4-dinitrophenylhydrazine (DNPH) [31]. In brief, cell lysates were incubated with 200 μL of 10 mM DNPH or HCl (2.5 M) for blank. After incubation for 1 h in the dark at 37 °C, proteins were precipitated with 20 % TCA and incubation on ice for 5 min, followed by centrifugation at 10,000 × *g* for 10 min at 4 °C. The protein pellets were washed three times with 1 mL of ethanol/ethyl acetate (1:1, v/v), dissolved in 6 M guanidine hydrochloride solution and incubated at 37 °C for 15 min. Carbonyl contents are detected at 370 nm on the basis of molar absorbance coefficient of 22000 M^−1^cm^−1^.

### Mitochondrial membrane potential (*ΔΨm*) assay

The change in the mitochondrial membrane potential (*ΔΨm*) in lung cancer cells was assessed using the fluorescent lipophilic cationic probe 5,5,6,6-tetrachloro-1,1,3,3-tetraethylbenzimidazolyl-carbocyanine iodide (JC-1) [32]. Cells were seeded into black 96-well titration plates. The antioxidant (NAC) was applied before F3 fraction application at 3 mM for 1 h. After 4 h of treatment with F3 fraction (½ IC_50_, IC_50_ and 2 IC_50_), cells were incubated with 10 μM of JC1 probe diluted in fresh medium at 37 °C for 30 min and then washed twice with PBS. The cellular fluorescence intensity of both JC-1 green monomers (excitation 485 nm, emission of 530 nm) and red dimers (excitation 530 nm, emission 590 nm) were measured on Victor *X*2 Multilabel Plate Reader (Perkin Elmer, USA). The results are shown as a ratio of red dimers to green monomers fluorescence in relation to the control fluorescence ratio, assumed to be 100 %.

### Estimation of antioxidants-related parameters

Cell lysates were used to measure superoxide dismutase (SOD) and catalase activities and reduced glutathione level (GSH) according to the procedures described elsewhere [33–35]. One SOD unit was defined as the amount of enzyme necessary to inhibit 50 % of the reaction rate and SOD activity was expressed as units of SOD per mg of protein. Catalase activity was expressed as micromoles of hydrogen peroxide consumed/min/mg protein and reduced glutathione level was expressed as μg GSH/mg protein using molar extinction coefficient of 13600 M^-1^ cm^-1^.

### Statistical analysis

All data were expressed as the means ± standard deviation (SD), from at least three independent experiments with similar results. Statistical analysis was performed by GraphPad Prism 5.01 (GraphPad software, Inc., USA). Experiments were analyzed using one-way ANOVA followed by Tukey post hoc test. Statistical significance is indicated *p ≤ 0.05, **p ≤ 0.01, and ***p ≤ 0.001.

## Results

### In vitro cytotoxicity of Aah venom and its toxic fractions

**Fig. 1:**
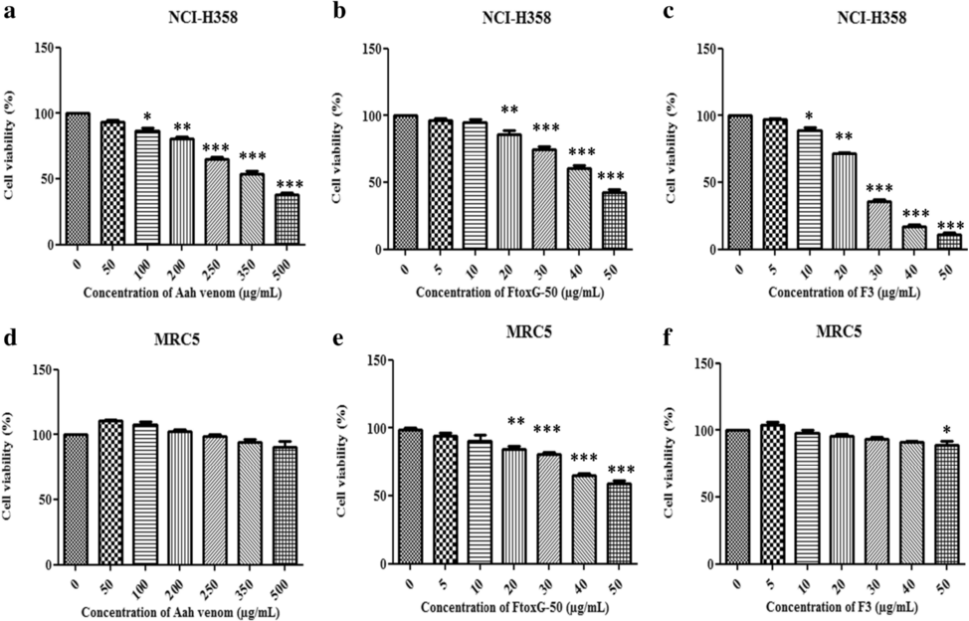
Dose-dependent effects of Aah venom and its toxic fractions (FtoxG-50 and F3) on the viability of NCI-H358 and MRC5 cells. Cells were treated with different concentrations of (**a**, **d**) Aah venom, (**b**, **e**) FtoxG-50 and (**c**, **f**) F3 fraction for 24 h. Cell viability was determined according to the MTT assay. The data are expressed as the mean ± SD of three independent experiments in triplicate. Significances are shown in comparison to control cells (*p < 0.05; **p < 0.01; ***p < 0.001)

Aah venom was tested for its cytotoxic effects on four different types of cell lines: HeLa, Hep2, MCF7 and NCI-H358 by MTT assay. The exposure to varying concentrations of Aah venom at 50, 100, 200, 250, 350 and 500 μg/mL significantly decreased the viability of NCI-H358 and MCF7 cells in a dose-dependent manner (Fig. 1, Table 1).

**Table 1:** IC_50_ values of Aah venom and cisplatin in panel of cell lines NCI-H358, MCF7, HeLa, Hep-2, and MRC5

Treatment	Unit	IC_50_				
		NCI-H358^a^	MCF-7^b^	HeLa^c^	Hep-2^d^	MRC5^e^
Aah venom	μg/mL	369.20 ± 1.33	458 ± 1.39	>500	>500	>500
Cisplatin	μM	11.2 ± 1.3	5.2 ± 1.6	6.31 ± 2.59	20 ± 1.8	9.3 ± 0.6

Cells were treated with Aah venom or positive control (cisplatin) at varying concentrations for 24 h and cell viability was determined by MTT assay. IC_50_ is the concentration of compound that inhibits cell growth by 50 % and values represent mean ± SD of three independent experiments

^a^NCI-H358: human lung adenocarcinoma cell line; ^b^MCF7: human breast cancer cell line; ^c^HeLa: human cervix adenocarcinoma; ^d^Hep-2: human laryngeal carcinoma cell line; ^e^MRC5: normal human lung fibroblast

NCI-H358 cell line was more sensitive to Aah venom (IC_50_ = 396.60 ± 1.33 μg/mL) than MCF7 (IC_50_ = 458 ± 1.39 μg/mL) after 24 h of exposure. No significant effect of the venom was observed on HeLa, Hep2 (data not shown) or normal lung fibroblast MRC5 cell lines viability at these concentrations (Fig. 1, Table 1). Comparatively, cisplatin revealed an inhibitory effect on NCI-H358, MCF-7, HeLa, Hep-2 cancer cells and MRC5 normal cells with different IC_50_ values (Table 1).

**Fig. 2:**
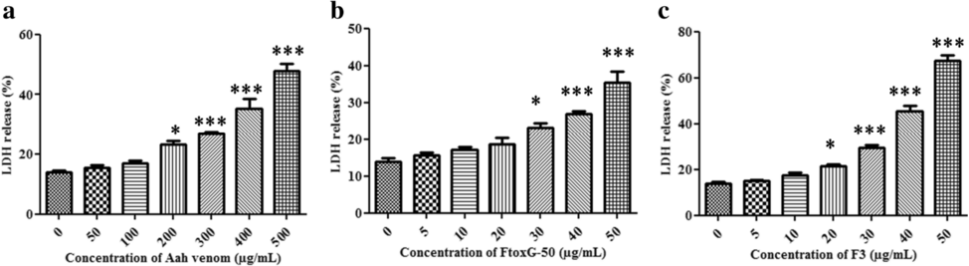
LDH release in NCI-H358 cells after 24 h of exposure to different concentrations of (**a**) Aah venom, (**b**) FtoxG-50 and (**c**) F3 fraction. The percent of LDH release was determined by dividing absorbance of the culture supernatant to absorbance of supernatant plus cell lysates. The data values are expressed as the mean ± SD of three independent experiments in triplicate. Indicated significances are shown in comparison to control (*p < 0.05; **p < 0.01; ***p < 0.001)

According to these results, the cytotoxic potential of Aah toxic fractions (FtoxG50 and F3) was evaluated only on the most sensitive cell line to the crude venom (NCI-H358 cells). Cell treatments with increasing concentration (5 to 50 μg/mL) of the two toxic fractions (FtoxG50 and F3) during 24 h resulted in a dose-dependent reduction of cell viability with and IC_50_ of 45.86 ± 0.91 μg/mL and 27.05 ± 0.70 μg/mL, respectively (Fig. 1). However, F3 fraction did not significantly affect normal human lung fibroblasts (MRC5) in comparison to FtoxG-50. LDH activity increased in dose-dependent manner in exposed cells to venom or toxic fractions for 24 h in comparison to untreated cells (Fig. 2). At the highest concentrations of Aah venom (500 μg/mL) or its fractions (50 μg/mL), LDH was elevated to 47.88 ± 4.14 %, 35.41 ± 5.38 % and 67.53 ± 3.14 % respectively compared to control group. The results of this cytotoxic assay were in correlation with MTT assay.

The most cytotoxic fraction seems to be F3 on lung cancer cells and its cytotoxic activity was 14-fold higher than Aah crude venom. The ½ IC_50_ (13.52 μg/mL), IC_50_ (27.05 μg/mL), 2 IC_50_ (54.1 μg/mL) concentrations of F3 fraction were selected to investigate the mechanism underlying its cytotoxicity in the subsequent experiments.

### Effect of F3 fraction on cell growth and cell morphology

**Fig. 3:**
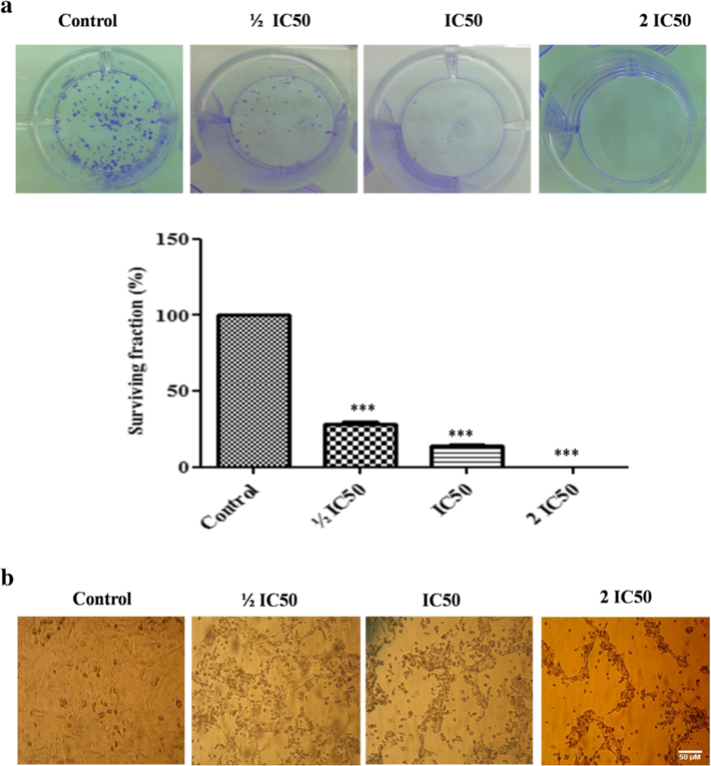
Morphological and cell growth assessment of NCI-H358 cells treated with F3 fraction. **a** Decrease of colony number and inhibition of surviving fraction after cell exposure to ½ IC_50_, IC_50_ and 2 IC_50_ concentrations of F3 fraction. The data are expressed as the mean ± SD of three independent experiments in triplicate. Significances are shown in comparison to control cells (***p < 0.001). **b** Morphological changes of NCI-H358 cells were observed under the phase contrast inverted microscope (magnification 100×) after the treatment with ½ IC_50_, IC_50_ and 2 IC_50_ concentrations of F3 fraction for 24 h

F3 fraction treatment resulted in a significant inhibition of colony formation of NCI-H358 cells in comparison to untreated cells. The percentage of surviving fraction shows that F3 fraction reduced colony formation to 28.19 ± 2.33 % and 13.94 ± 1.92 % at ½ IC_50_ and IC_50_ concentration, respectively, with a complete inhibition of cell proliferation at 2 IC_50_ concentration (Fig. 3a). Indeed, exposure of NCI-H358 cells to different concentrations of F3 fraction for 24 h induced morphological changes in a dose-dependent manner and exhibited alterations in cell monolayer with areas devoid of cells, rupture of membranes and release of cytosolic contents and cell shrinkage (Fig. 3b).

### Effect of F3 fraction on apoptotic morphological changes

**Fig. 4:**
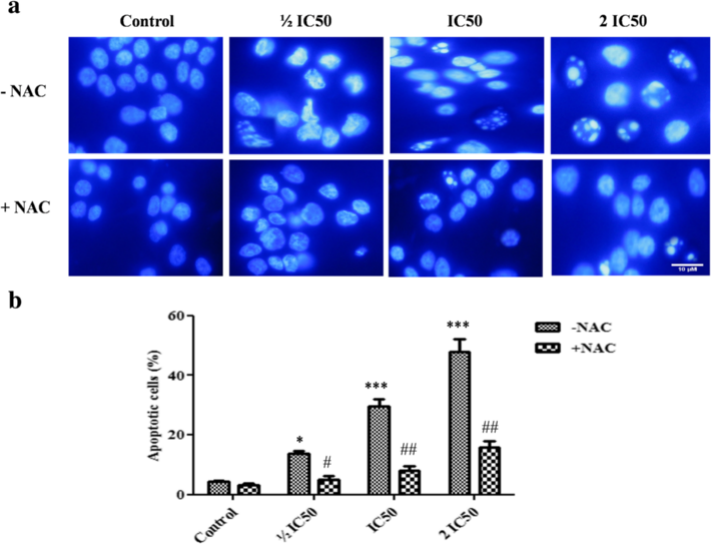
Apoptosis induced by F3 fraction. **a** Apoptotic morphology changes of NCI-H358 lung cells 24 h after F3 fraction treatment (½ IC_50_, IC_50_ and 2 IC_50_) in the absence (–NAC) or presence (+NAC) of N-acetylcysteine. Cells were stained with Hoechst 33258 and visualized by fluorescence microscopy (Zeiss Axioplan, Germany; magnification 400×). **b** Percentage of apoptotic cells induced by F3 fraction with or without NAC pre-treatment (3 mM for 1 h). Significances are shown in comparison to control and to the preincubated cells with NAC for each F3 fraction concentration (*p < 0.05; **p < 0.01; ***p < 0.001; vs. control and ^#^ *p* < 0.05; ^##^ *p* < 0.01, vs. the corresponding dose of F3 fraction with NAC pre-treatment)

Figure 4a shows the morphological changes of NCI-H358 cells after 24 h of incubation with F3 fraction (½ IC_50_, IC_50_ and 2 IC_50_) in the absence (–NAC) or presence (+NAC) of ROS scavenger. The quantitative data obtained by analysis of images are presented in Fig. 4b. The nuclei of untreated cells appeared to be in intact oval shape and with less bright blue fluorescence. Treated cells with increasing concentrations of F3 fraction, on the other hand, exhibited morphological changes that are typical of apoptosis such as cellular nucleus shrinkage, chromatin condensation, formation of apoptotic bodies and cell decrement. After 24 h, the number of apoptotic cells enhanced significantly (*p* ≤ 0.05; *p* < 0.001), being 3.07-fold, 6.71-fold and 10.80-fold of that of the control after exposure to ½ IC_50_, IC_50_ and 2 IC_50_ concentrations, respectively. Apoptosis was significantly reversed by pretreatment with antioxidant NAC. However, NAC treatment alone did not affect apoptosis.

### Effect of F3 fraction on DNA fragmentation

**Fig. 5:**
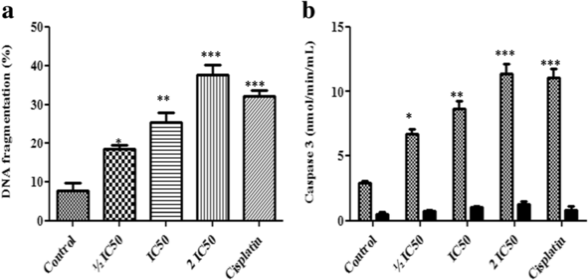
Effect of F3 fraction on the percentage of DNA fragmentation and caspase-3 activity. **a** Effect of F3 fraction on percentage of DNA fragmentation in NCI-H358 cells. **b** Caspase-3 activity in NCI-H358 cells was analyzed using CASP-3-C colorimetric assay kit. Cells were treated with F3 fraction (½ IC_50_, IC_50_ and 2 IC_50_) for 24 h with (black bars) or without (grey bars) caspase-3 inhibitor Ac-DEVD-CHO. The results are expressed as the mean ± SD of triplicate determinations, representative of three independent experiments. Significances indicated were in comparison to control (*p < 0.05; **p < 0.01; ***p < 0.001 vs. control)

F3 fraction treatment induced a significant increase in DNA fragmentation in a concentration-dependent manner. It was enhanced by 2.38-, 3.29- and 4.87-fold for ½ IC_50_, IC_50_ and 2 IC_50_ concentrations respectively while by 4.17-fold due to treatment at IC_50_ concentration of cisplatin in comparison to DNA from untreated cells after 24 h of incubation (Fig. 5a).

### Effect of F3 fraction on caspase-3 activity

In cells treated with F3 fraction, caspase-3 activity significantly increased to 2.32-, 3.00- and 3.94- fold for ½ IC_50_, IC_50_ and 2 IC_50_ concentrations, respectively, and 3.34 fold increases for IC_50_ of cisplatin as compared to control. The specific contribution of caspase-3 activity was confirmed by using the inhibitor Ac-DEVD-CHO that significantly inhibits caspase-3 activity at all F3 fraction concentrations (Fig. 5b).

### Effect of F3 fraction on intracellular ROS generation

**Fig. 6:**
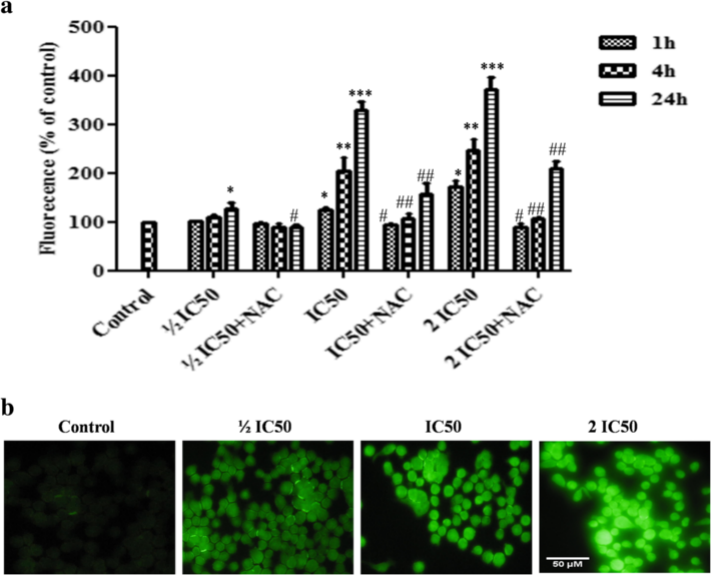
Intracellular ROS generation in absence (–NAC) or presence (+NAC) of N-acetylcysteine. **a** Quantitative intracellular DCF fluorescence intensity of NCI-H358 cells induced by F3 fraction with or without the presence of NAC. The data are expressed as means ± SD from three independent experiments. Significances are shown in comparison to control and to the preincubated cells with NAC for each F3 fraction dose (*p < 0.05; **p < 0.01; ***p < 0.001; vs. control and ^#^ *p* < 0.05; ^##^ *p* < 0.01, vs. the corresponding dose of F3 fraction with NAC pre-treatment). **b** Microscopic images of ROS generation in NCI-H358 cells after treatment with different concentrations of F3 fraction (½ IC_50_, IC_50_ and 2 IC_50_) for 4 h

The results showed an increase in ROS production in exposed cells to F3 fraction in a dose and time-dependent manner (Fig. 6a). Cell treatment with a low dose of F3 fraction (½ IC_50_) did not significantly affect ROS production after 1 or 4 h of exposure. However, in cells treated with increasing concentrations of F3 fraction (IC_50_ and 2 IC_50_), ROS production was significantly increased at all time treatments. At the highest concentration (2 IC_50_), F3 fraction enhanced the ROS levels approximately two and three-folds with respect to untreated cells at both 4 h and 24 h treatment. As shown in Fig. 6b, the green fluorescence intensity augmented gradually with increasing concentrations of F3 fraction after 4 h of exposure. The content of ROS was significantly (*p* ≤ 0.05; *p* ≤ 0.0 1) reduced in NAC pretreated group compared to the F3 fraction treated group (½ IC_50_, IC_50_ and 2 IC_50_) and was almost completely restored after 1 h and 4 h for all treatment concentrations (Fig. 6a).

### Effect of F3 fraction on nitric oxide production, lipid peroxidation and protein oxidation

**Fig. 7:**
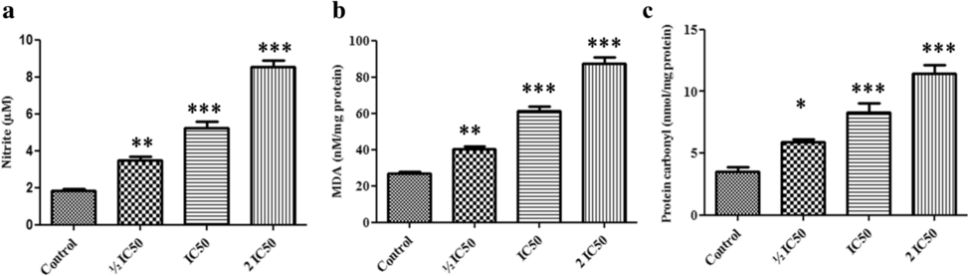
Effect of F3 fraction on levels of **a** nitrite, **b** MDA and **c** protein carbonyl products in NCI-H358 cells. The results are expressed as the mean ± SD of triplicate determinations, representative of three independent experiments. Significances indicated were in comparison to control (*p < 0.05; **p < 0.01; ***p < 0.001 vs. control)

As shown in Fig. 7a, the production of NO was measured by analyzing its stable product (nitrite) in culture medium of control and F3 fraction treated cells. F3 fraction enhanced production of NO in NCI-H358 cells by significantly increasing the nitrite level in a dose-dependent manner to 1.92-fold (½ IC_50_), 2.77-fold (IC_50_) and 4.69-fold (2 IC_50_) compared to controls.

NCI-H358 cells exposed to ½ IC_50_, IC_50_ and 2 IC_50_ concentrations of F3 fraction for 24 h showed that intracellular MDA levels, a product of membrane lipid peroxidation, were significantly higher than in unexposed cells (Fig. 7b). It was increased to 1.46, 2.26 and 3.22 for each concentration, respectively, when compared to the control cells.

As shown in Fig. 7c, the level of protein carbonyls, a biomarker of protein oxidation, was measured in NCI-H358 cells treated with F3 fraction at ½ IC_50_, IC_50_ and 2 IC_50_ concentrations during 24 h. Protein carbonyls levels were elevated to 1.66, 2.35 and 3.24 fold respectively compared to untreated cells.

### Effect of F3 fraction on mitochondrial membrane potential (*ΔΨm*)

**Fig. 8:**
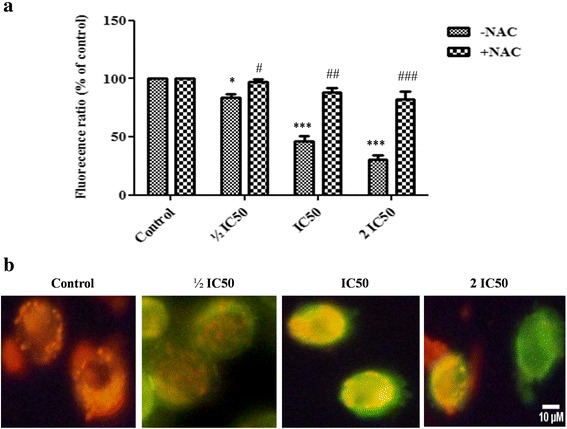
Changes of mitochondrial membrane potential (Δ*Ψm*) induced by F3 fraction in NCI-H358 cells. **a** Concentration-dependent reduction of red/green fluorescence ratio corresponding to loss of Δ*Ψm* in NCI-H358 cells elicited by increasing concentration of F3 fraction (½ IC_50_, IC_50_ and 2 IC_50_) for 4 h. The results are expressed as the mean ± SD of triplicate determinations, representative of three independent experiments. Significances indicated were in comparison to control cells and to the preincubated cells with NAC for each F3 fraction dose (*p < 0.05; ***p < 0.001 vs. control cells and ^#^ *p* < 0.05; ^##^ *p* < 0.01; ^###^ *p* < 0.001, vs. the corresponding dose of F3 fraction with NAC pre-treatment). **b** Microscopic images of Δ*Ψm* dissipation in NCI-H358 cells after 4 h of treatment with F3 fraction (½ IC_50_, IC_50_ and 2 IC_50_)

In lung cancer cells, the ratio of red/green dye of JC-1 probe was significantly decreased to 83.41 ± 3.86 %, 46.22 ± 6.64 % and 30.14 ± 7.55 % at 4 h after treatment with F3 fraction at ½ IC_50_, IC_50_ and 2 IC_50_ concentrations, respectively, in comparison to control cells (Fig. 8 a, b). This indicates that F3 fraction induced depolarization of *ΔΨm* in NCI-H358 cells during apoptosis. In order to ascertain whether ROS were involved in the alteration of *ΔΨm,* the cells were preincubated with NAC before treatment with different concentrations of F3 fraction. As shown in Fig. 8a, the level of Δ*Ψm* was nearly similar to control at ½ IC_50_. However, NAC treatment did not completely inhibit the fraction F3-induced loss of Δ*Ψm* at IC_50_ and 2 IC_50_ concentrations.

### Effect of F3 fraction on intracellular enzymatic and non-enzymatic antioxidants

**Fig. 9:**
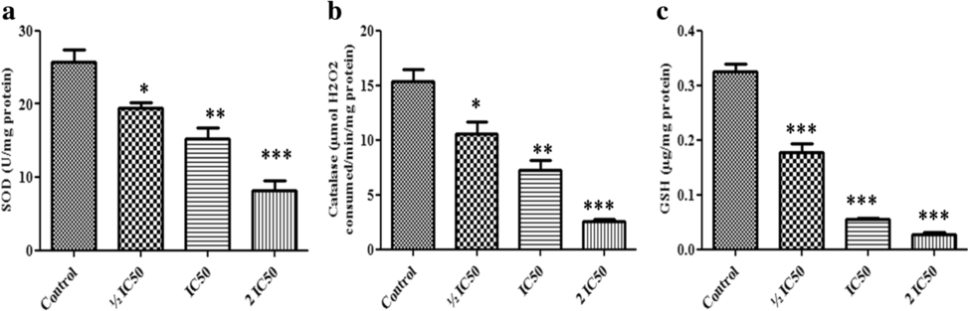
Effect of F3 fraction on enzymatic (**a**) SOD, (**b**) catalase and non-enzymatic antioxidants status (**c**) glutathione (GSH) in NCI-H358 cells. The results are expressed as the mean ± SD of triplicate determinations, representative of three independent experiments. Significances indicated were in comparison to control (*p < 0.05; **p < 0.01; ***p < 0.001 vs. control)

The antioxidant response to F3 fraction cells exposure was evaluated by SOD and catalase activities and the level of cellular non-enzymatic tripeptide GSH on NCI-H358 cells (Fig. 9a, b, c). The obtained results showed that F3 fraction lowered the activity of SOD to 75.68 %, 59.30 % and 31.65 % while they markedly reduced catalase activity to 69.03 %, 47.65 % and 16.42 % in comparison to the control group. The cellular GSH content were also significantly decreased in a dose-dependent manner. GSH level was reduced to 48.33 %, 16.66 % and 11.66 % after treatment with ½ IC_50_, IC_50_ and 2 IC_50_ concentrations of F3 fraction respectively.

## Discussion

Continuous efforts have been made in recent years in the search for new agents to improve the treatment against cancer. Several bioactive components from biological products (such as proteins, peptides and enzymes) that can induce apoptosis in cancer cells have the potential to become suitable candidates as anticancer drugs. In this sense, peptides obtained from scorpion venoms have proven to be valuable tools for the development of new drugs [1, 2].

The current study demonstrated that Aah scorpion venom significantly reduced proliferation of NCI-H358 cells in a dose-dependent manner. The non-small cell lung cancer cell line NCI-H358 was the most sensitive to Aah venom in comparison to MCF7, Hep2 and HeLa cell lines. The variations in response to crude venom on growth of the four cell types after 24 h exposure might be a result of the complex composition of the venom and/or the targets expressed on cancer cells [12]. This observation is consistent with previous studies in which venoms of other scorpions such as *Rhopalurus junceus*, *Androctonus crassicauda* and *Centruroides limpidus limpidus* also induced selective and differential cytotoxicity against different human malignant cell lines [12, 36, 37].

According to these results, the cytotoxic potential of Aah toxic fractions (FtoxG-50 and F3) was assessed only on the most sensitive cell line to the crude venom (NCI-H358). Cell treatment with increasing concentrations of the toxic fractions (FtoxG-50 and F3) during 24 h resulted in a dose-dependent drop of cell viability. The F3 fraction showed more cytotoxic effect towards NCI-H358 cells than FtoxG-50. Interestingly, F3 fraction did not significantly affect the normal human lung fibroblast compared to toxic fraction FtoxG-50. The difference in the cytotoxic effect between the two tested fractions on lung cancer cells and normal fibroblastic cells could be due to their different composition and/or different cellular targets.

Indeed, accumulated evidence has indicated that several Kv channel subtypes are widely expressed in the plasma membranes of numerous cells and that these channels are involved both in the regulation of proliferation and apoptosis [38, 39]. It was reported that margatoxin, a selective Kv1.3 blocker, inhibited the cell proliferation of human lung adenocarcinoma A549 cell line by cell cycle arrest in G1 phase [40]. Jang et al. [41] showed cell growth inhibition in A549 cells after exposure to dendrotoxin-k, a Kv1.1 blocker, without affecting cell proliferation of normal MRC5. These authors reported the very low expression of Kv1.1 mRNA and protein in MRC-5 cells compared to A549 cells. Therefore, in the present study, the F3 fraction seems to induce a selective cytotoxicity on NCI-H358 lung cancer cells without any significant effect on normal lung fibroblast suggesting the overexpression of Kv channels on the plasma membranes of NCI-H358 cells.

Additionally, cytotoxicity events were confirmed by the increase of LDH leakage in the Aah venom and toxic fraction-treated cells which is usually correlated with loss of cell membrane integrity [42]. In order to assess the molecular mechanism underlying F3 fraction induced-cytotoxicity, ½ IC_50_ (13.52 μg/mL), IC_50_ (27.05 μg/mL) and 2 IC_50_ (54.1 μg/mL) concentrations were selected for the subsequent experiments.

Besides the obtained cytotoxicity, F3 fraction induced inhibition of cell proliferation after testing over a longer treatment period with the clonogenic assay in a dose-dependent manner. Cells exposed to different concentrations of F3 fraction exhibited morphological changes evidenced by: alterations in cell monolayer with areas devoid of cells, rupture of membranes and release of cytosolic contents. We also observed round and shrunk cells, which are morphological features of apoptosis. Similar morphological alterations were observed after treatment with fraction FI isolated from *Tityus discrepans* scorpion venom on human breast carcinoma cell line SKBR3 [15].

Apoptosis is a highly regulated process that occurs in almost all living organisms that selectively eliminates transformed cells. Consequently, the induction of apoptosis by cytotoxic natural compounds is considered as the main key and efficient strategy for cancer therapy and new drug development [26, 43]. To elucidate whether F3 fraction inhibits the proliferation of NCI-H358 cells by inducing apoptosis, treated cells were examined after staining with the DNA binding fluorochrome Hoechst 33258 and were analyzed for DNA fragmentation and caspase-3 activity. Our results showed that cells exposed to increasing concentrations of F3 fraction displayed nuclear morphological alterations which are indicative of apoptosis characterized by chromatin condensation, DNA fragmentation, cell shrinkage and compartmentalization of the dead cells into apoptotic bodies associated with the decrease of cell viability.

Breakdown of the nucleus is a hallmark of apoptosis that occurs during the early phase of this mode of cell death [26]. This includes the condensation of chromatin and associated fragmentation of the DNA followed by breakdown of the entire nucleus. The caspase family plays an important role in the initiation and execution pathways of cell apoptosis, in which caspase-3 acts as the central protease regulator and is required for the progression of apoptosis that is activated during the cascade [44, 45]. It was observed that caspase-3 activity was significantly increased in F3 fraction treated cells and inhibited by its specific inhibitor Ac-DEVD-CHO. All these results suggest that F3 fraction has an apoptosis-inducing effect on NCI-H358 cells, which is supported by several studies that reported the ability of some venoms from scorpions and spiders to trigger apoptosis through DNA fragmentation and caspase-3 activation [14, 36, 45–47]. Furthermore, NAC, (a general free radical scavenger) significantly reduced the F3 fraction-induced apoptosis in NCI-H358. Therefore, we can infer that reactive oxygen species (ROS) may be involved in the F3 fraction-mediated apoptotic process.

ROS are the key mediators of cellular oxidative stress and have an important role in tumor cell damage and mitochondrial stability. Imbalance redox status of ROS levels may harm major cellular components such as DNA, proteins, lipids and membranes leading to oxidative damage and cell death [48]. To determine whether the apoptosis of NCI-H358 cells was induced by ROS production, we quantified intracellular ROS by DCF fluorescence after cell exposure to ½ IC_50_, IC_50_ and 2 IC_50_ concentrations of F3 fraction for 1, 4 and 24 h. The obtained results showed an increase in ROS production in exposed cells to F3 fraction in dose and time-dependent manner with significant increase after 24 h of treatment for all tested concentrations (1.27, 3.29, 3.71 fold increase at ½ IC_50_, IC_50_ and 2 IC_50_ concentrations respectively). Moreover, NAC significantly inhibited the intracellular ROS production, which was completely counteracted at 1 h and 4 h. This result suggests that ROS may be a key early signal of F3 fraction-induced apoptosis. Similar to our present results, it has been reported that toxins from various animal venoms could induce intrinsic apoptosis through ROS upregulation, a situation that could be prevented by pretreatment with antioxidants [43, 49–54].

Our data showed also that F3 fraction enhanced the production of nitrite, a primary product of NO metabolism in lung cancer cells. It has been previously demonstrated that nitric oxide can increase the oxidative stress by the production of reactive nitrogen species (RNS), which are a variety of nitrogen containing molecules that are typically derived via NO reactions [55]. Excessive RNS generation contribute to biomembrane damage including mitochondrial membrane and in the formation of permeability transition pore [46, 47, 55]. High levels of NO can also alter protein functions through S-nitrosylation and/or nitration of regulatory proteins and increased Fas density on some tumor cell surface such as CaP cells or SW480 human colon carcinoma cells [55, 56]. Earlier studies also reported the involvement of nitrosative stress in the venoms induced apoptosis [46, 47].

Malondialdehyde is a final metabolite of lipid peroxidation and was formed from a variety of unsaturated fatty acids in biological membranes stimulated by ROS and RNS overproduction [57]. Protein-bound carbonyls represent a marker of overall protein oxidation, as they are formed early during oxidative stress conditions in blood, tissues and cells [48, 57, 58]. In the present study, we found that F3 fraction could increase the MDA and protein carbonyl levels in the NCI-H358 cells in a dose-dependent manner. These results clearly indicate that lipid peroxidation of cell membranes and protein oxidation were induced in response to ROS and RNS generation after F3 fraction treatment. Enhancement of these stable peroxidation products could be an essential factor of apoptosis increasing cellular oxidative stress [44, 58, 59].

Mitochondrial membrane potential (*ΔΨm*) is a key parameter for many mitochondrial functions including ion transport, ATP production and ROS generation [48]. On the other hand, disruption of *ΔΨm* promotes mitochondrial dysfunction, oxidative damage and various apoptotic processes [60]. In the present study, loss of mitochondrial membrane potential (Δ*Ψm*) was evaluated in NCI-H358 by using the cationic lipophilic dye, JC-1, which has been widely used to detect alteration of mitochondrial membrane integrity considered as one of the early events of apoptosis. We observed a dose-dependent decrease in *ΔΨm* after 4 h of treatment with ½ IC_50_, IC_50_ and 2 IC_50_ concentrations of F3 fraction. The alteration of *ΔΨm* was indicative of the involvement of the mitochondria in the apoptotic processes induced by F3 fraction. Previous studies also reported the involvement of *ΔΨm* alteration in venom induced mitochondria damage and cell death [14, 43, 46, 47, 52, 53]. Moreover, the collapse of the *ΔΨm* was attenuated, but not completely abolished, by NAC pretreatment which suggests that F3 fraction induced *ΔΨm* collapse with further ROS generation resulted from mitochondrial membrane damage. Similar results were obtained with cardiotoxin 3 from the cobra *Naya naya atra* and *Pelagia noctiluca* crude venom on neuroblastoma cells [49, 53].

High levels of oxidative damage can be caused by not only oxidative stress, but also by the dysfunction of the cellular repair system, which may modify the cell defense system, provoking cell death [57, 58]. Cellular antioxidant defense systems including SOD, catalase, and GSH may prevent disturbances in ROS homeostasis, or reduce the effect of oxidative stress in cells [44, 57]. Thus, we further investigated the cellular antioxidant defense systems in F3 fraction treated cells by assessment of such antioxidants. Our results showed that F3 fraction significantly reduces the activity of cellular antioxidant enzymes such as SOD and catalase, and significantly depleted intracellular GSH. The alteration in the antioxidant system could reflect the excessive reactive oxygen and nitrogen species overproduction and oxidative damage due to F3 fraction cells treatment. Previous reports also indicated that *Odontobuthus doriae* venom and plancitoxin I isolated from the venom of crown-of-thorns starfish *Acanthaster planci* reduce the cellular antioxidant level in response to high oxidative stress [44, 46].

**Fig. 10:**
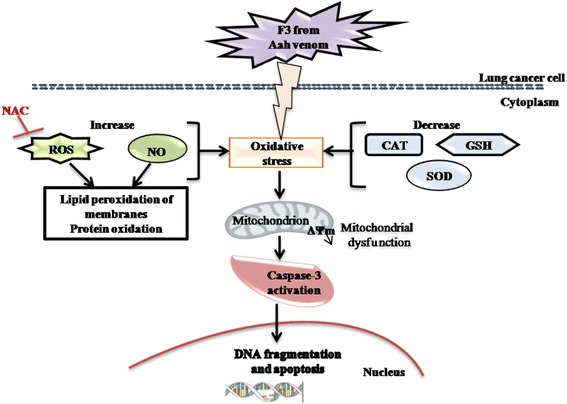
Schematic diagram showing the mechanism by which toxic F3 fraction of *Androctonus australis hector* venom could induce apoptosis in non-small cell lung cancer NCI-H358

The present findings suggest that the F3 fraction exhibits a potent ability to promote ROS generation in NCI-H358 cells by eliciting oxidative stress and depleting cellular antioxidants (SOD, catalase, GSH) (Fig. 10). This dual property is a promising approach to make the tumor cells more vulnerable to further oxidative stress induced by exogenous ROS-generating agents such as F3 fraction. Currently, several anticancer therapeutic agents are known to stimulate oxidative stress and thus kill tumor cells in a preferential manner [48]. Besides, the F3 fraction-induced mitochondrial dysfunction – as a consequence of excessive ROS production and *ΔΨm* disruption (Fig. 10) – would modulate the opening of the mitochondrial permeability transition pore resulting in release of cytochrome *c* and other pro-apoptotic factors from the mitochondrial intermembrane space into cytosol, and then trigger to caspase cascades activation and apoptosis [60].

## Conclusion

In summary, this study reports that F3 fraction exhibits potent cytotoxic and antiproliferative effects on NCI-H358 cells. This fraction seems to trigger apoptotic cell death on lung cancer cells through the activation of mitochondrial-mediated pathway along with reactive oxygen and nitrogen species production, depletion in antioxidant defense states and depolarization of mitochondrial membrane. Similarly to the non-toxic fraction F1, the toxic fraction F3 displayed anti-tumoral potential in vitro, which could be exploited in the development of new therapeutic agents.

## Abbreviations

Aah: *Androctonus australis hector*
F1: non-toxic fraction purified from Aah venom
FtoxG50 and F3: toxic fractions purified from Aah venom
GSH: Glutathione or γ-glutamyl-cysteinyl-glycine
IC_50_: Inhibitory concentration 50
K_v_: potassium voltage gated channels
LDH: Lactate dehydrogenase
MDA: Malondialdehyde
NAC: N-acetylcysteine
Na_v_: sodium voltage gated channels
NO: Nitric oxide
RNS: Reactive nitrogen species
ROS: Reactive oxygen species
SOD: Superoxide dismutase
*ΔΨm*: Mitochondrial membrane potential

## References

[1] Ortiz E, Gurrola GB, Schwartz EF, Possani LD (2015). Scorpion venom components as potential candidates for drug development. Toxicon.

[2] Ding J, Chua PJ, Bay BH, Gopalakrishnakone P (2014). Scorpion venoms as a potential source of novel cancer therapeutic compounds. Exp Biol Med (Maywood).

[3] Heinen TE, da Veiga AB (2011). Arthropod venoms and cancer. Toxicon.

[4] Gomes A, Bhattacharjee P, Mishra R, Biswas AK, Dasgupta SC, Giri B (2010). Anticancer potential of animal venoms and toxins. Indian J Exp Biol.

[5] Cordeiro FA, Amorim FG, Anjolette FAP, Arantes EC (2015). Arachnids of medical importance in Brazil: main active compounds present in scorpion and spider venoms and tick saliva. J Venom Anim Toxins incl Trop Dis.

[6] Sartim MA, Sampaio SV (2015). Snake venom galactoside-binding lectins: a structural and functional overview. J Venom Anim Toxins incl Trop Dis.

[7] Costa TR, Burin SM, Menaldo DL, de Castro FA, Sampaio SV (2014). Snake venom L-amino acid oxidases: an overview on their antitumor effects. J Venom Anim Toxins incl Trop Dis.

[8] Wang WX, Ji YH (2005). Scorpion venom induces glioma cell apoptosis in vivo and inhibits glioma tumor growth in vitro. J Neurooncol.

[9] Zargan J, Sajad M, Umar S, Naime M, Ali S, Khan HA (2011). Scorpion (*Odontobuthus doriae*) venom induces apoptosis and inhibits DNA synthesis in human neuroblastoma cells. Mol Cell Biochem.

[10] Das Gupta S, Debnath A, Saha A, Giri B, Tripathi G, Vedasiromoni JR (2007). Indian black scorpion (*Heterometrus bengalensis Koch*) venom induced antiproliferative and apoptogenic activity against human leukemic cell lines U937 and K562. Leuk Res.

[11] Almaaytah A, Tarazi S, Mhaidat N, Al-Balas Q, Mukattash TL (2013). Mauriporin, a Novel Cationic α-Helical Peptide with Selective Cytotoxic Activity Against Prostate Cancer Cell Lines from the Venom of the Scorpion *Androctonus mauritanicus*. Int J Pept Res Ther.

[12] Caliskan F, Ergene E, Sogut I, Hatipoglu I, Basalp A, Sivas H (2013). Biological assays on the effects of Acra3 peptide from Turkish scorpion *Androctonus crassicauda* venom on a mouse brain tumor cell line (BC3H1) and production of specific monoclonal antibodies. Toxicon.

[13] Deshane J, Garner CC, Sontheimer H (2003). Chlorotoxin Inhibits Glioma Cell Invasion via Matrix Metalloproteinase-2. J Biol Chem.

[14] Das Gupta S, Gomes A, Debnath A, Saha A, Gomes A (2010). Apoptosis induction in human leukemic cells by a novel protein Bengalin, isolated from Indian black scorpion venom: Through mitochondrial pathway and inhibition of heat shock proteins. Chem Biol Interact.

[15] D’Suze G, Rosales A, Salazar V, Sevcik C (2010). Apoptogenic peptides from *Tityus discrepans* scorpion venom acting against the SKBR3 breast cancer cell line. Toxicon.

[16] Guo X, Ma C, Du Q, Wei R, Wang L, Zhou M (2013). Two peptides, TsAP-1 and TsAP-2, from the venom of the Brazilian yellow scorpion, *Tityus serrulatus*: Evaluation of their antimicrobial and anticancer activities. Biochimie.

[17] Shao JH, Cui Y, Zhao MY, Wu CF, Liu YF, Zhang JH (2014). Purification, characterization, and bioactivity of a new analgesic-antitumor peptide from Chinese scorpion *Buthus martensii Karsch*. Peptides.

[18] Laraba-Djebari F, Adi-Bessalem S, Hammoudi-Triki D, Gopalakrishnakone P, Possani LD, Schwartz EF, de la Vega RC R (2015). Scorpion Venoms: Pathogenesis and Biotherapies. Scorpion Venoms.

[19] Hammoudi-Triki D, Ferquel E, Robbe-Vincent A, Bon C, Choumet V, Laraba-Djebari F (2004). Epidemiological data, clinical admission gradation and biological quantification by ELISA of scorpion envenomations in Algeria: effect of immunotherapy. Trans R Soc Trop Med Hyg.

[20] Adi-Bessalem S, Hammoudi-Triki D, Laraba-Djebari F (2015). Scorpion Venom Interactions with the Immune System. Scorpion Venoms.

[21] Laraba-Djebari F, Hammoudi-Triki D (1998). Use of toxic fraction isolated from Algerian *Androctonus australis hector* scorpion venom for the assessment of anti-venom serum. Arch Inst Pasteur Alger.

[22] Martin-Eauclaire MF, Legros C, Bougis PE, Rochat H. Les toxines des venins de scorpion. Ann Inst Pasteur Actual. 1999;10:207–22.

[23] Bekkari N, Laraba-Djebari F (2015). Beneficial effects of *Androctonus australis hector* venom and its non-toxic fraction in the restoration of early hepatocyte-carcinogenesis induced by FB1mycotoxin: Involvement of oxidative biomarkers. Exp Mol Pathol.

[24] Bradford MM (1976). A Rapid and sensitive method for the quantitation of microgram quantities of protein utilizing the principle of protein-dye binding. Anal Biochem.

[25] Carmichael J, DeGraff WG, Gazdar AF, Minna JD, Mitchell JB (1987). Evaluation of a tetrazolium-based semiautomated colorimetric assay: assessment of chemosensitivity testing. Cancer Res.

[26] Henry CM, Hollville E, Martin SJ (2013). Measuring apoptosis by microscopy and flow cytometry. Methods.

[27] Bouaziz C, Abid-Essefi S, Bouslimi A, El Golli E, Bacha H (2006). Cytotoxicity and related effects of T-2 toxin on cultured Vero cells. Toxicon.

[28] Wang H, Joseph JA (1999). Quantifying cellular oxidative stress by dichlorofluorescein assay using microplate reader. Free Radic Biol Med.

[29] Ding AH, Nathan CF, Stuehr DJ (1988). Release of reactive nitrogen intermediates and reactive oxygen intermediates from mouse peritoneal macrophages. Comparison of activating cytokines and evidence for independent production. J Immunol.

[30] Ohkawa H, Ohishi N, Yagi K (1979). Assay for lipid peroxides in animal tissues by thiobarbituric acid reaction. Anal Biochem.

[31] Levine RL, Garland D, Oliver CN, Amici A, Climent I, Lenz AG (1990). Determination of carbonyl content in oxidatively modified proteins. Methods Enzymol.

[32] Nuydens R, Novalbos J, Dispersyn G, Weber C, Borgers M, Geerts H (1999). A rapid method for the evaluation of compounds with mitochondria-protective properties. J Neurosci Methods.

[33] Beauchamp C, Fridovich I (1971). Superoxide dismutase: improved assays and an assay applicable to acrylamide gels. Anal Biochem.

[34] Sinha AK (1972). Colorimetric assay of catalase. Anal Biochem.

[35] Sedlak J, Lindsay RH (1968). Estimation of total, protein-bound, and nonprotein sulfhydryl groups in tissue with Ellman’s reagent. Anal Biochem.

[36] Díaz-García A, Morier-Díaz L, Frión-Herrera Y, Rodríguez-Sánchez H, Caballero-Lorenzo Y, Mendoza-Llanes D (2013). In vitro anticancer effect of venom from Cuban scorpion *Rhopalurus junceus* against a panel of human cancer cell lines. J Venom Res.

[37] Contreras-Ortiz JM, Vázquez-Chagoyán JC, Martínez-Castañeda JS, Estrada-Franco JG, Aparicio-Burgos JE, Acosta-Dibarrat J (2013). Resistance of cervical adenocarcinoma cells (HeLa) to venom from the scorpion *Centruroides limpidus limpidus*. J Venom Anim Toxins incl Trop Dis.

[38] Comes N, Bielanska J, Vallejo-Gracia A, Serrano-Albarrás A, Marruecos L, Gómez D (2013). The voltage-dependent K+ channels Kv1.3 and Kv1.5 in human cancer. Front Physiol.

[39] Urrego D, Tomczak AP, Zahed F, Stuhmer W, Pardo LA (2014). Potassium channels in cell cycle and cell proliferation. Philos Trans R Soc Lond B Biol Sci.

[40] Jang SH, Choi SY, Ryu PD, Lee SY (2011). Anti-proliferative effect of Kv1.3 blockers in A549 human lung adenocarcinoma in vitro and in vivo. Eur J Pharmacol.

[41] Jang SH, Ryu PD, Lee SY (2011). Dendrotoxin-κ suppresses tumor growth induced by human lung adenocarcinoma A549 cells in nude mice. J Vet Sci.

[42] Decker T, Lohmann-Matthes L (1998). A quick and simple method for the quantitation of lactate dehydrogenase release in measurements of cellular cytotoxicity and tumor necrosis factor (TNF) activity. J Immunol Methods.

[43] Lee CC, Hsieh HJ, Hsieh CH, Hwang DF (2014). Spine venom of crown-of-thorns starfish (*Acanthaster planci*) induces antiproliferation and apoptosis of human melanoma cells (A375.S2). Toxicon.

[44] Lee CC, Hsieh HJ, Hsieh CH, Hwang DF (2015). Plancitoxin I from the venom of crown-of-thorns starfish (*Acanthaster planci*) induces oxidative and endoplasmic reticulum stress associated cytotoxicity in A375.S2 cells. Exp Mol Pathol.

[45] Liu Z, Zhao Y, Li J, Xu S, Liu C, Zhu Y (2012). The venom of the spider *Macrothele raveni* induces apoptosis in the myelogenous leukemia K562 cell line. Leuk Res.

[46] Zargan J, Umar S, Sajad M, Naime M, Ali S, Khan HA (2011). Scorpion venom (*Odontobuthus doriae*) induces apoptosis by depolarization of mitochondria and reduces S-phase population in human breast cancer cells (MCF-7). Toxicol In Vitro.

[47] Zargan J, Sajad M, Umar S, Naime M, Ali S, Khan HA (2011). Scorpion (*Androctonus crassicauda*) venom limits growth of transformed cells (SH-SY5Y and MCF-7) by cytotoxicity and cell cycle arrest. Exp Mol Pathol.

[48] Kardeh S, Ashkani-Esfahani S, Alizadeh AM (2014). Paradoxical action of reactive oxygen species in creation and therapy of cancer. Eur J Pharmacol.

[49] Morabito R, Condello S, Currò M, Marino A, Ientile R, La Spada G (2012). Oxidative stress induced by crude venom from the jellyfish *Pelagia noctiluca* in neuronal-like differentiated SH-SY5Y cells. Toxicol In Vitro.

[50] Park MH, Jo M, Won D, Song HS, Han SB, Song MJ (2012). Snake venom toxin from *Vipera lebetina turanica* induces apoptosis of colon cancer cells via upregulation of ROS-and JNK-mediated death receptor expression. BMC Cancer.

[51] Zhang L, Wei LJ (2007). ACTX-8, a cytotoxic L-amino acid oxidase isolated from *Agkistrodon acutus* snake venom, induces apoptosis in Hela cervical cancer cells. Life Sci.

[52] Tu WC, Wu CC, Hsieh HL, Chen CY, Hsu SL (2008). Honeybee venom induces calcium-dependent but caspase-independent apoptotic cell death in human melanoma A2058 cells. Toxicon.

[53] Chen KC, Lin SR, Chang LS (2008). Involvement of mitochondrial alteration and reactive oxygen species generation in Taiwan cobra cardiotoxin-induced apoptotic death of human neuroblastoma SK-N-SH cells. Toxicon.

[54] Lee CC, Hsieh HJ, Hwang DF (2015). Cytotoxic and apoptotic activities of the plancitoxin I from the venom of crown-of-thorns starfish (*Acanthaster planci*) on A375.S2 cells. J Appl Toxicol.

[55] Bonavida B, Khineche S, Huerta-Yepez S, Garbán H (2006). Therapeutic potential of nitric oxide in cancer. Drug Resist Updat.

[56] Jeannin JF, Leon L, Cortier M, Sassi N, Paul C, Bettaieb A (2008). Nitric oxide-induced resistance or sensitization to death in tumor cells. Nitric Oxide.

[57] Valko M, Leibfritz D, Moncol J, Cronin MT, Mazur M, Telser J (2007). Free radicals and antioxidants in normal physiological functions and human disease. Int J Biochem Cell Biol.

[58] Melegari SP, Perreault F, Moukha S, Popovic R, Creppy EE, Matias WG (2012). Induction to oxidative stress by saxitoxin investigated through lipid peroxidation in Neuro 2A cells and Chlamydomonas reinhardtii alga. Chemosphere.

[59] Ayed Y, Boussabbeh M, Zakhama W, Bouaziz C, Abid S, Bacha H (2011). Induction of cytotoxicity of *Pelagia noctiluca* venom causes reactive oxygen species generation, lipid peroxydation induction and DNA damage in human colon cancer cells. Lipids Health Dis.

[60] Caroppi P, Sinibaldi F, Fiorucci L, Santucci R (2009). Apoptosis and human diseases: mitochondrion damage and lethal role of released cytochrome C as proapoptotic protein. Curr Med Chem.

